# Reconstitution of Targeted Deadenylation by the Ccr4-Not Complex and the YTH Domain Protein Mmi1

**DOI:** 10.1016/j.celrep.2016.10.066

**Published:** 2016-11-15

**Authors:** James A.W. Stowell, Michael W. Webster, Alexander Kögel, Jana Wolf, Kathryn L. Shelley, Lori A. Passmore

**Affiliations:** 1 MRC Laboratory of Molecular Biology, Cambridge CB2 0QH, UK

**Keywords:** gene expression, poly(A) tail, Ccr4-Not, exonuclease, RNA

## Abstract

Ccr4-Not is a conserved protein complex that shortens the 3′ poly(A) tails of eukaryotic mRNAs to regulate transcript stability and translation into proteins. RNA-binding proteins are thought to facilitate recruitment of Ccr4-Not to certain mRNAs, but lack of an in-vitro-reconstituted system has slowed progress in understanding the mechanistic details of this specificity. Here, we generate a fully recombinant Ccr4-Not complex that removes poly(A) tails from RNA substrates. The intact complex is more active than the exonucleases alone and has an intrinsic preference for certain RNAs. The RNA-binding protein Mmi1 is highly abundant in preparations of native Ccr4-Not. We demonstrate a high-affinity interaction between recombinant Ccr4-Not and Mmi1. Using in vitro assays, we show that Mmi1 accelerates deadenylation of target RNAs. Together, our results support a model whereby both RNA-binding proteins and the sequence context of mRNAs influence deadenylation rate to regulate gene expression.

## Highlights

• We purify a fully recombinant *S. pombe* Ccr4-Not complex
• The complex has an intrinsic substrate preference, and both Ccr4 and Caf1 are active
• Ccr4-Not stably interacts with the YTH domain RNA-binding protein Mmi1
• Mmi1 accelerates deadenylation of RNAs by Ccr4-Not in a sequence-specific manner

## Graphical Abstract

**Figure:**
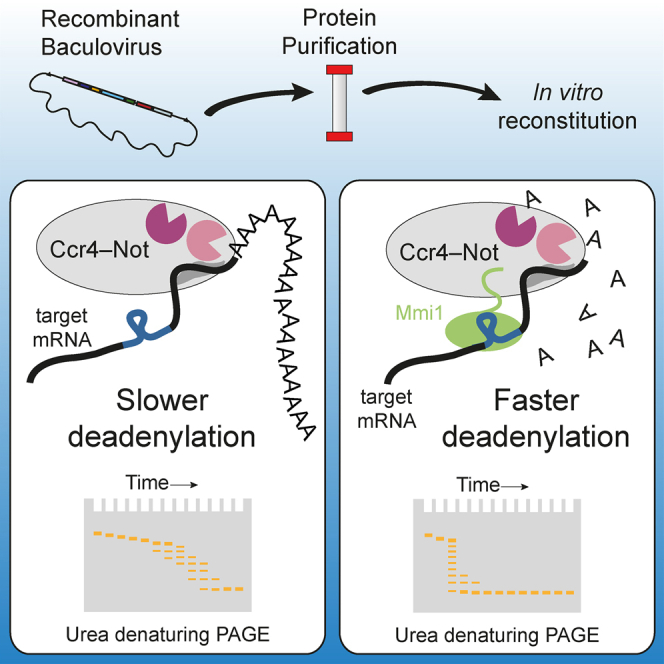

## Teaser

Poly(A) tails regulate mRNA stability and translation. Stowell et al. show that the rate of poly(A) tail removal by a fully recombinant Ccr4-Not complex is influenced by both the substrate RNA sequence and an RNA-binding adapter protein, Mmi1.

## Introduction

Ccr4-Not is a conserved multi-protein complex that regulates gene expression at multiple levels. It influences transcription, mRNA stability, and translation (Collart, 2016), playing important roles in numerous cellular pathways including cell cycle, early development, inflammation, and neuronal processes (Beilharz and Preiss, 2007, Lackner et al., 2007, Weill et al., 2012). Ccr4-Not possesses exonuclease activity that shortens the poly(A) tail found at the 3′ end of almost every eukaryotic mRNA, a process called deadenylation (Tucker et al., 2001). This initiates cytoplasmic mRNA decay (Decker and Parker, 1993, Garneau et al., 2007). Poly(A) tails are also important for efficient translation of mRNAs into proteins (Goldstrohm and Wickens, 2008, Kapp and Lorsch, 2004), and the length of the poly(A) tail correlates with the efficiency of translation initiation in some situations (Subtelny et al., 2014).

**Figure 1:**
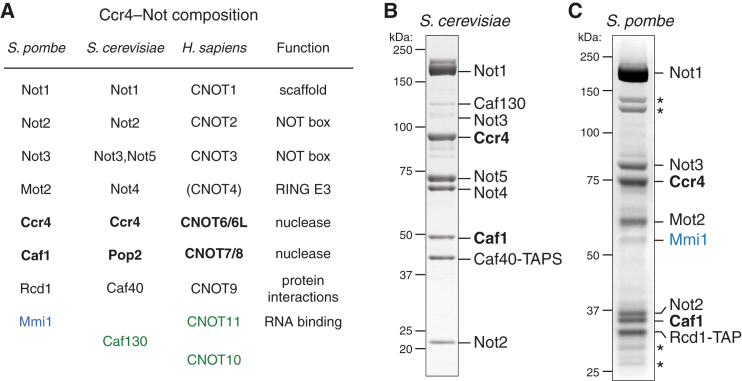
Purification of Native Ccr4-Not Complexes (A) Table of Ccr4-Not subunits from fission yeast (*S. pombe*), budding yeast (*S. cerevisiae*), and human. CNOT4, in parentheses, is not stably associated with the human complex. Subunits listed in green are not conserved across all eukaryotes. Mmi1 (blue) is a specificity factor (RNA-binding protein) that co-purifies with the complex. Exonuclease subunits are in bold. (B and C) Ccr4-Not was purified from *S. cerevisiae* (B) and *S. pombe* (C) strains containing a TAP-tagged Caf40/Rcd1 subunit. The purifications were analyzed by SDS-PAGE and stained with Coomassie blue. Bands were identified using mass spectrometry. Asterisks mark degradation products of core subunits. See also Figure S1.

Ccr4-Not contains seven core subunits, including two exonucleases, Caf1 (also called Pop2, CNOT7, or CNOT8) and Ccr4 (also called CNOT6 or CNOT6L) (Collart, 2016, Tucker et al., 2001, Wahle and Winkler, 2013) (Figure 1A). Caf1 and Ccr4 interact with each other directly (Basquin et al., 2012), but the relevance of two different nucleases within the complex is unclear. Caf1 and several other subunits bind the Not1 protein that is thought to act as a scaffold (Basquin et al., 2012, Petit et al., 2012). The other subunits include Not2, Not3, and, in some species, Not5, which contain a conserved NOT box motif (Bhaskar et al., 2013, Boland et al., 2013). They promote mRNA decapping to co-ordinate downstream steps in mRNA degradation (Alhusaini and Coller, 2016). Not4 (also called Mot2 or CNOT4) contains a RING finger E3 ubiquitin ligase (Bhaskar et al., 2015) and is not stably associated with the other subunits in some species. Rcd1 (also called Caf40 or CNOT9) is highly conserved, but little is known about its role within the complex.

Targeting of particular mRNAs for deadenylation by Ccr4-Not is thought to be mediated by adapter proteins, which bind specific sequence elements, often in the 3′ UTR (Wahle and Winkler, 2013). For example, tristetraprolin (TTP), a regulator of the inflammatory response, binds to AU-rich elements to facilitate rapid turnover of the mRNAs containing them (Fabian et al., 2013, Lykke-Andersen and Wagner, 2005). Similarly, the RNA-binding protein Nanos recruits Ccr4-Not to play key roles in mRNA regulation during early development (Bhandari et al., 2014, Raisch et al., 2016, Suzuki et al., 2012), while Puf/Pumilio proteins target specific RNAs in diverse processes, including stem cell maintenance (Goldstrohm et al., 2006, Hook et al., 2007, Miller and Olivas, 2011). The GW182/TNRC6 subunit of the microRNA-induced silencing complex (miRISC) recruits Ccr4-Not to mediate translational repression and/or mRNA decay of microRNA targets (Braun et al., 2011, Chekulaeva et al., 2011, Chen et al., 2014, Fabian et al., 2011, Mathys et al., 2014).

Ccr4-Not has been challenging to reconstitute in vitro because of the number of subunits and their large sizes (e.g., Not1 is 200–250 kDa, depending on the species). Here, we characterize the composition of the fission yeast complex and generate a fully recombinant Ccr4-Not using baculovirus-mediated insect cell expression. This complex is active at removing poly(A) tails from substrate RNAs but does not substantially degrade the upstream 3′ UTRs. We also demonstrate that the *Schizosaccharomyces pombe* RNA-binding protein Mmi1 binds stably to recombinant Ccr4-Not. We show that Mmi1 accelerates the deadenylation of specific target RNAs in vitro, supporting a model where RNA-binding proteins act as adapters to generate specificity in RNA degradation that is triggered by Ccr4-Not.

## Results

### Purification of Native Yeast Ccr4-Not Complexes

To investigate the biochemical activity and specificity of Ccr4-Not, we required purified proteins. Thus, we isolated the endogenous complex from *Saccharomyces cerevisiae* using a tandem affinity purification (TAP) approach. Preparations from a yeast strain where the Caf40 subunit was TAP tagged yielded a purified Ccr4-Not complex with all nine subunits, as assessed by SDS-PAGE and mass spectrometry (Figure 1B). Both Caf130 and Not3 were present in substoichiometric amounts. This heterogeneity would complicate the interpretation of biochemical assays.

We examined the sequences of the protein subunits and found that many of the *S. cerevisiae* orthologs contain glutamine/asparagine-rich stretches, often in amino-terminal extensions. These are particularly prominent in Ccr4 and Caf1 (Figure S1). Interestingly, the described subunits of fission yeast Ccr4-Not are generally shorter and lack many of the Gln/Asn-rich regions found in the budding yeast complex, making the entire complex substantially smaller and potentially more compact (540 kDa compared to 802 kDa). Since this could facilitate purification and stability, we purified the endogenous complex from fission yeast.

Using a *S. pombe* yeast strain carrying a C-terminal TAP tag on the endogenous *rcd1* gene, which encodes a core Ccr4-Not subunit, we obtained a seven-subunit complex (Figure 1C). We confirmed the identities of all seven conserved core subunits with mass spectrometry, including a Not4 ortholog, which is also stably bound in *S. cerevisiae* but not in *H. sapiens* (Figure 1A) (Bai et al., 1999, Lau et al., 2009). We did not identify any orthologs of Caf130, CNOT10, or CNOT11, which are found in some other eukaryotic Ccr4-Not complexes. Several degradation products of the core subunits could also be detected.

Surprisingly, the RNA-binding protein Mmi1 was highly abundant in *S. pombe* Ccr4-Not preparations and was visible on Coomassie-blue-stained gels (Figure 1C). This interaction was not sensitive to RNase treatment, consistent with Mmi1 binding being independent of RNA.

### Production of a Recombinant Ccr4-Not Complex

In vitro analyses of the deadenylation activity of Ccr4-Not have been limited by the complexity of the reagents required. Previous studies have used small quantities of endogenous complex that are not fully homogeneous (Goldstrohm et al., 2006, Goldstrohm et al., 2007, Jeske et al., 2006). Similarly, the yield and purity of our preparations of endogenous Ccr4-Not from *S. cerevisiae* or *S. pombe* were inadequate for detailed biochemical analyses.

Other studies on Ccr4-Not activity used recombinant nuclease subcomplexes (e.g., Fabian et al., 2013, Hook et al., 2007, Jonstrup et al., 2007, Maryati et al., 2015, Wang et al., 2010). However, pull-down studies and crystal structures containing short fragments of the RNA-binding proteins TTP and Nanos reveal that they both interact with the Ccr4-Not scaffold protein Not1 (Bhandari et al., 2014, Fabian et al., 2013). Hence, studies on nuclease subcomplexes will not reveal how the exonucleases are coordinated with RNA-binding activities—a crucial property of the intact complex.

**Figure 2:**
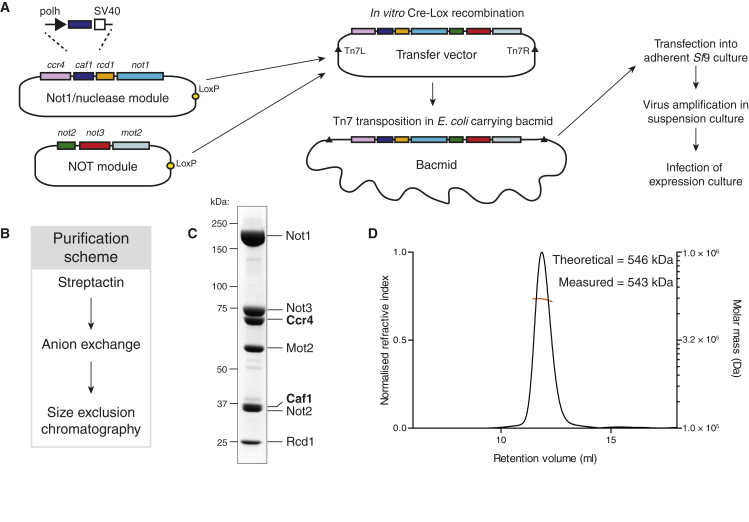
Production of Recombinant Ccr4-Not (A) Scheme for the cloning and expression of a recombinant Ccr4-Not complex. The seven core subunit gene cassettes (with polyhedrin promoters and SV40 [simian virus 40] terminators) were cloned into one of two separate vectors before being combined by in vitro Cre-Lox recombination. The resulting vector was stably transposed into the baculovirus genome for downstream recombinant virus production and protein expression in the *Sf*9 insect cell line. (B) Protein purification scheme. (C) SDS-PAGE analysis of purified Ccr4-Not, demonstrating the high purity obtained by recombinant expression, as well as the uniform stoichiometry among constituent subunits. (D) Analysis of purified recombinant Ccr4-Not by size exclusion chromatography with multi-angle light scattering (SEC-MALS) reveals that it is monodisperse. The normalized refractive index and theoretical and experimental molecular masses (including affinity tags) are shown. See also Figure S2A.

To overcome these limitations, we developed an overexpression and purification strategy to isolate a fully recombinant Ccr4-Not. We chose to purify the fission yeast complex due to its smaller size and reduced low-complexity (Asn/Gln) sequence. We used the MultiBac system (Bieniossek et al., 2012) to co-express all seven core subunits (defined by our preparations of native complex; Figure 1C) from a single baculovirus in *Sf*9 cells (Figure 2A). A single affinity step using a StrepII tag on Caf1, followed by anion exchange and size exclusion chromatography in native conditions yielded approximately 1 mg pure Ccr4-Not complex per liter of culture (Figures 2B and 2C). The purified complex contains all seven subunits. Examination of the band intensities suggests that all subunits are present in equal stoichiometry. Tandem mass spectrometry analysis of the purified sample showed that there are no contaminating insect cell subunits or exonucleases (Figure S2A). Size exclusion chromatography coupled with multi-angle light scattering (SEC-MALS) demonstrated that the purified complex has a molecular weight of ∼0.5 MDa and is monodisperse in solution (Figure 2D). This is in agreement with the theoretical mass of the seven subunits in unit stoichiometry.

### Recombinant Ccr4-Not Is an Active Deadenylase

**Figure 3:**
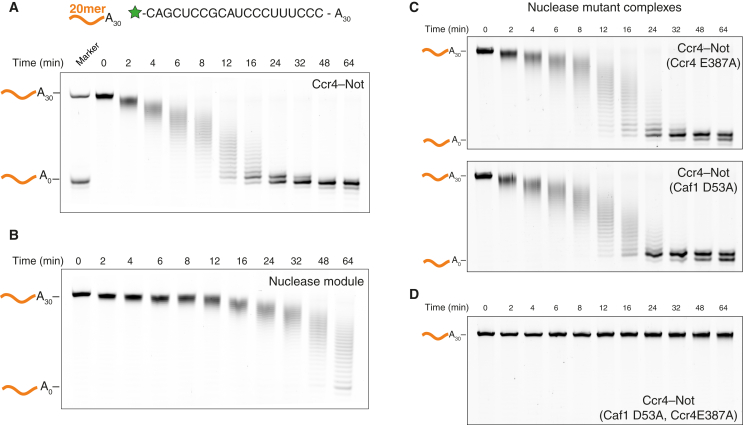
Recombinant Ccr4-Not Specifically Removes Poly(A) Tails In Vitro (A) Deadenylation of an unstructured RNA substrate with 30 3′ adenosines (20-mer-A_30_) by recombinant *S. pombe* Ccr4-Not complex. The reaction was analyzed by denaturing PAGE. The sizes of the RNA substrate with and without the poly(A) tail are shown. The sequence of the 20-mer model RNA is shown above, with a green star representing the fluorescein fluorophore. See also Figures S2B–S2E and S3A. (B) Deadenylation of the 20-mer-A_30_ substrate with recombinant Caf1-Ccr4 nuclease module. See also Figure S4. (C and D) Deadenylation assays with recombinant Ccr4-Not complexes containing point mutations in the active sites of (C) Caf1 (D53A), Ccr4 (E387A), or (D) both. See also Figures S2–S4.

To measure the deadenylation activity of recombinant Ccr4-Not, we tested its ability to remove a 3′ poly(A) tail from a model RNA. We used a synthetic 5′ fluorescently labeled substrate comprising a 20-mer RNA followed by 30 adenosines (20-mer-A_30_; Figure 3A). A similar RNA substrate has been used previously to study the in vitro activity of Caf1 and is predicted to be unstructured (Figure S3A) (Jonstrup et al., 2007). We assayed changes in the length of the poly(A) tail with denaturing PAGE.

Recombinant Ccr4-Not was active and efficiently removed the 3′ poly(A)_30_ tail in less than 24 min (Figure 3A). It did not substantially degrade the RNA upstream of the poly(A) tail. This activity was not dependent on the length of the upstream segment since a poly(A)_30_ RNA lacking an upstream region was fully degraded (Figure S2B). Furthermore, a substrate containing the unstructured 20-mer sequence followed by ten adenosines (20-mer-A_10_) is deadenylated at a rate similar to that of the 20-mer-A_30_ substrate (Figures S2C–S2E). Thus, our fully recombinant Ccr4-Not complex, comprising seven different protein subunits, is an active deadenylase in vitro, where the exonuclease activity is specific for the 3′ poly(A) tail.

### Intact Ccr4-Not Is More Active Than the Exonucleases Alone

We next compared the activity of the full recombinant complex to that of the nucleases alone. The two exonuclease subunits Ccr4 and Caf1 have been shown to interact directly through the leucine-rich repeat (LRR) domain of Ccr4 (Basquin et al., 2012). We purified the Caf1-Ccr4 heterodimeric complex and found that it migrates as a single species using size exclusion chromatography (Figure S4). We tested the recombinant Caf1-Ccr4 complex for the ability to remove a 30-mer poly(A) tail using the deadenylation assay described earlier. Compared with the intact complex, the nucleases have substantially reduced exonuclease activity and do not completely deadenylate the substrate in 64 min (Figure 3B). Thus, binding of Ccr4 and Caf1 to the complex confers increased activity, likely by stabilizing or activating the nucleases or through the contribution of other subunits, e.g., by RNA-binding activity.

### Ccr4-Not Contains Two Active Exonucleases

To determine the contributions of each of the two nucleases to deadenylation within the context of the entire complex, we cloned, co-expressed, and purified complexes that contained single-point mutations in either Caf1 (D53A) or Ccr4 (E387A). These mutations have been shown to disrupt the co-ordination of magnesium in the active sites, rendering the nucleases inactive (Jonstrup et al., 2007, Wang et al., 2010). Intriguingly, when we tested the deadenylation activity using the same 20-mer-A_30_ substrate as described earlier, there was little change in the activity of either mutant complex compared to wild-type (Figure 3C). Both Caf1 and Ccr4 can thus act as exonucleases within the entire Ccr4-Not complex. The activity of a complex with inactivating point mutations in both nucleases is abolished (Figure 3D), confirming that the observed nuclease activity is not due to contaminants.

### Recombinant Ccr4-Not Forms a Stable and Stoichiometric Complex with Mmi1

To understand how Ccr4-Not selectively targets certain transcripts, we wished to test the effect of an RNA-binding protein on deadenylation. In our preparations of native Ccr4-Not, a substantial quantity of Mmi1 co-purified (Figure 1C). Mmi1 (meiotic mRNA interceptor 1) is an RNA-binding protein essential to the fission yeast *S. pombe* (Harigaya et al., 2006). Many meiotic genes, including the transcription factor *mei4* and the meiotic cohesin subunit *rec8*, are transcribed during vegetative growth but selectively eliminated. This depends on Mmi1 and *cis*-acting RNA elements in target transcripts known as DSRs (determinants of selective removal). Repression of these transcripts likely occurs through multiple mechanisms, including heterochromatin formation and RNA decay (Harigaya et al., 2006, Hiriart et al., 2012, McPheeters et al., 2009, Sugiyama and Sugioka-Sugiyama, 2011, Zofall et al., 2012). Upon entry into meiosis, Mmi1 is sequestered by binding a series of DSR elements in the long non-coding RNA *meiRNA* (Harigaya et al., 2006, Shichino et al., 2014, Yamashita et al., 2012). This stabilizes meiotic transcripts, restoring their expression and resulting in large changes in transcriptional and post-transcriptional programs (Cremona et al., 2011, Mata et al., 2002).

**Figure 4:**
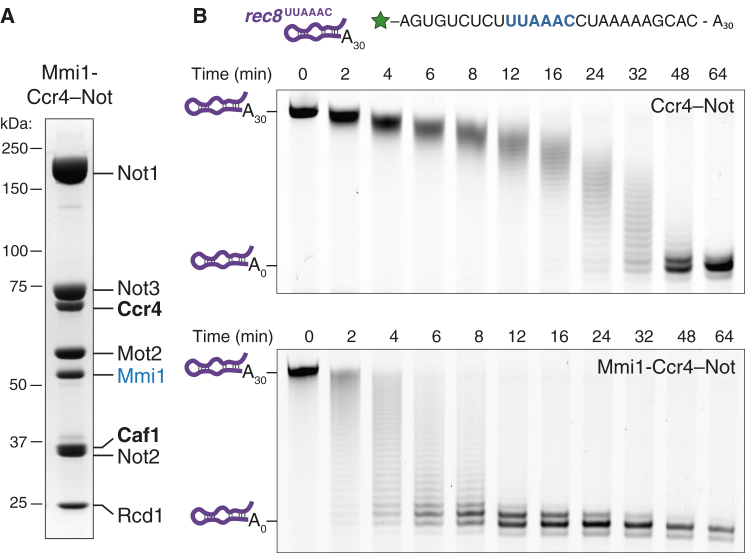
Mmi1 Forms a Complex with Ccr4-Not and Accelerates Deadenylation on Target RNAs (A) SDS-PAGE analysis of recombinant Ccr4-Not co-purified with Mmi1. (B) Deadenylation of a target RNA by recombinant Ccr4-Not without (upper gel) and with (lower gel) bound Mmi1. RNA substrate length was monitored by denaturing PAGE. The sizes of the RNA substrates with and without the poly(A) tail are shown—both include a 26-nt upstream region. The sequence of this Mmi1 target RNA substrate is shown above and contains a DSR motif from the *rec8* 3′ UTR followed by 30 3′ adenosines. The green star represents the fluorescein fluorophore. See also Figures S3–S5.

To determine whether recombinant Ccr4-Not binds Mmi1 like the native complex, we generated a new expression strategy to combine Mmi1 with the fully assembled Ccr4-Not in a single recombinant baculovirus expressing all eight genes (Figure S5A). Using the same purification procedure described earlier, Mmi1 co-purified with the complex in approximately stoichiometric quantities (Figure 4A). Thus, Mmi1 binds Ccr4-Not stably and with sufficient affinity to remain associated throughout the entire purification.

### Mmi1 Accelerates Deadenylation of Target RNAs by Ccr4-Not

Mmi1 contains a YTH domain—an RNA-binding domain found in a variety of eukaryotic proteins (Stoilov et al., 2002, Zhang et al., 2010). Recent studies have shown that many YTH domains are readers of the highly abundant N^6^-methyladenosine (m^6^A) RNA modification (Dominissini et al., 2012, Luo and Tong, 2014, Yue et al., 2015). However, Mmi1 recognizes a hexanucleotide UNAAAC motif within DSRs and is not specific for m^6^A (Harigaya et al., 2006, Kilchert et al., 2015, Wang et al., 2016, Yamashita et al., 2012).

Mmi1 is the only RNA-binding protein that co-purified with native Ccr4-Not in large quantities (Figure 1C). Since Mmi1 interacts with specific RNAs and with Ccr4-Not, it could act as a model for the numerous specificity factors that target certain RNAs.

Thus, to investigate the molecular mechanisms by which intact Ccr4-Not deadenylates specific RNAs, we tested the activity of Mmi1-bound Ccr4-Not. We used an RNA substrate containing 26 nt of a *rec8* DSR (*rec8*^UUAAAC^-A_30_), an in vivo target of Mmi1 (Figures 4B and S3B) (Harigaya et al., 2006). Mmi1 substantially accelerates deadenylation of this target RNA substrate (Figure 4B). The rate of deadenylation is increased by ∼5-fold (Figure S5D). In addition, Mmi1 appears to increase the processivity of Ccr4-Not, since completely deadenylated product appears before the disappearance of RNAs with long poly(A) tails (Figure 4B, 4 min). These experiments suggest that Mmi1 facilitates Ccr4-Not recruitment to target RNAs, likely by tethering them to the complex, and could act as a paradigm for specificity factors.

### Ccr4-Not Has an Intrinsic Preference for Certain RNAs

Unexpectedly, apo Ccr4-Not has reduced activity on the DSR-containing *rec8*^UUAAAC^-A_30_ RNA substrate relative to the unstructured 20-mer-A_30_ (∼2-fold; Figures S5B and S5C). The *rec8*^UUAAAC^-A_30_ RNA is predicted to form a hairpin (Figure S3B). Furthermore Ccr4-Not shows slightly reduced activity on the poly(A)_30_ RNA lacking an upstream region (Figure S2E). This suggests that the sequence or secondary structure upstream of the poly(A) tail influences the activity of Ccr4-Not directly, independent of the presence of additional RNA-binding proteins.

**Figure 5:**
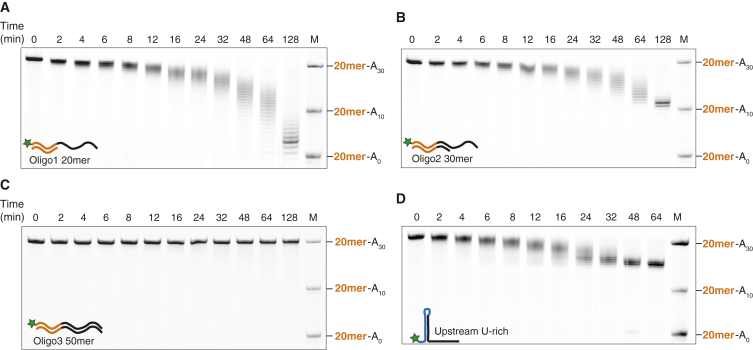
Ccr4-Not Deadenylation Activity Is Reduced on Duplex Nucleic Acid Substrates (A–C) Deadenylation by a recombinant Ccr4-Not complex of the 20-mer-A_30_ RNA substrate annealed to (A) a 20-nt DNA oligo complementary to the region upstream of the poly(A)_30_ tail; (B) a 30-nt DNA oligo; or (C) a 50-nt DNA oligo complementary to the entire 20-mer-A_30_ RNA. A control reaction performed on the 20-mer-A_30_ RNA in the same experiment is in Figures S6A and S6B. (D) Deadenylation of an RNA substrate that contains an upstream polyuridine stretch that is predicted to form a stable hairpin-loop structure with the poly(A) tail. See also Figures S3D, S6C, and S6D. M, molecular weight marker consisting of the 20-mer substrate with 0, 10, or 30 As. See also Figures S3 and S6.

To test this, we designed DNA oligos that anneal to the 20-mer-A_30_ RNA and tested the ability of Ccr4-Not to deadenylate these heteroduplex substrates (Figure 5). Relative to the single-stranded 20-mer-A_30_ RNA, Ccr4-Not had an impaired deadenylation rate when the 20 nt upstream of the A_30_ tail were base paired to an oligonucleotide (compare Figure 5A to Figure S6A). The deadenylation rate was similar when the duplex region was increased to 30 nt (including 10 nt of the polyA tail) but the complex is halted at the start of the heteroduplex (Figures 5B and S6B). Deadenylation is abrogated when the entire substrate is made a heteroduplex (Figure 5C).

In vivo, such duplex regions could arise if U-rich sequences are upstream of the poly(A) tail. To test Ccr4-Not activity on such a substrate, we designed a 53-mer RNA comprising a 23-nt U-rich region upstream of a 30-nt poly(A) tail (Figure S3D). Ccr4-Not activity was reduced on this U-rich-A_30_ RNA, compared to the unstructured 20-mer-A_30_ substrate, and it was unable to remove the entire A_30_ tail (Figures 5D, S6C, and S6D).

Taken together, these data show that Ccr4-Not cannot unwind nucleic acid duplexes that would arise due to base pairing of the poly(A) tail with U-rich sequences. In addition, Ccr4-Not has an intrinsic preference for single-stranded substrates, suggesting that it may bind single-stranded RNA directly, independent of RNA specificity factors.

### Mmi1 Accelerates Deadenylation in a Sequence-Specific Manner

In our assays, Mmi1 accelerated Ccr4-Not deadenylation of DSR-containing RNA to a greater extent than the unrelated non-target 20-mer-A_30_ RNA. Still, the deadenylation rate of Mmi1-Ccr4-Not (compared to apo Ccr4-Not) is slightly increased on the non-target unstructured 20-mer-A_30_ RNA (Figures S5B and S5D).

**Figure 6:**
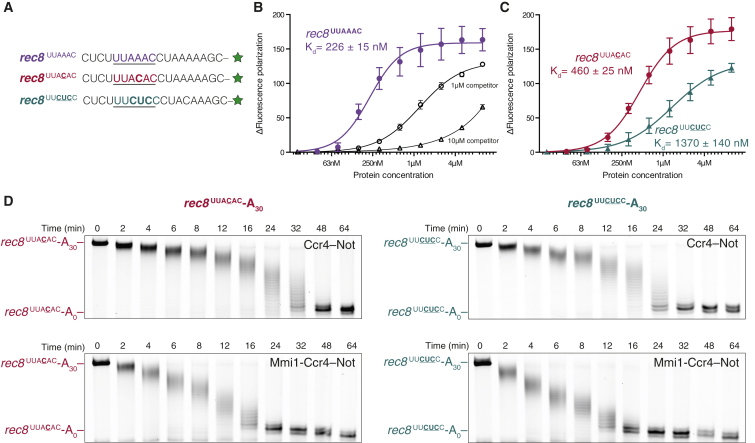
The DSR Sequence Is Required for Mmi1 Acceleration of Deadenylation (A–C) Fluorescence polarization experiments assaying binding of the YTH domain of Mmi1 to DSR RNAs. (A) RNA sequences with the hexanucleotide DSR sequence underlined. Green stars represent the fluorescein fluorophore. Mutated nucleotides are in bold. (B) The purified Mmi1 YTH construct specifically binds a DSR motif from the *rec8* mRNA. Binding experiments in the presence of the same unlabeled RNA at the indicated concentrations are plotted in black. (C) Mutations of the central adenosines within the DSR motif reduce the affinity of the interaction. Plots in (B) and (C) show the change in fluorescence polarization signal of 10 nM fluorescently labeled RNA upon addition of purified protein. Error bars represent the SD of five independent experiments. (D) Deadenylation assays with recombinant Ccr4-Not (top panels) and Mmi1-Ccr4-Not (bottom panels) complexes show that mutation of either a single adenosine (left) or all three adenosines (right) within the DSR of the model substrate reduces the ability of Mmi1 to accelerate deadenylation by Ccr4–Not. See also Figures S3, S6E, and S7.

To test the sequence specificity of this activity, we first purified a construct containing the Mmi1 YTH domain (without its N-terminal low-complexity region) to characterize its RNA-binding properties (Figure S7A). We determined the dissociation constant (K_D_) for the Mmi1-RNA interaction by measuring the change in fluorescence polarization signal of a DSR RNA from the *rec8* 3′ UTR carrying a 3′ fluorescein label (*rec8*^UUAAAC^; Figure 6A). This revealed a strong interaction with a K_D_ of 226 ± 15 nM (Figure 6B). Furthermore this interaction is specific because an unlabeled *rec8*^UUAAAC^ RNA acts as a competitor to disrupt binding (Figure 6B, black curves).

We evaluated the sequence specificity of this interaction by measuring the change in fluorescence polarization with DSR RNAs containing sequence substitutions. Previous work had shown that the As in positions 3, 4, and 5 of the DSRs are almost invariant (Kilchert et al., 2015, Yamashita et al., 2012). We found that pyrimidine substitution of the central adenosines decreased the affinity of Mmi1 YTH for DSR RNA: substituting a single central adenosine decreases the affinity ∼2-fold, whereas substituting all three As to pyrimidines decreases the affinity ∼6-fold (Figure 6C). In comparison, substitutions in the second or sixth position have little effect on the K_D_, while the 20-mer unstructured RNA binds weakly, with a K_D_ of ∼14 μM (Figures S7B and S7C).

These affinity measurements showed that changes in the central As of the DSR sequence impair the binding of Mmi1. We tested whether reduced affinity for the RNA substrate affects accelerated deadenylation by Mmi1 using in vitro deadenylation assays. Interestingly, we found that the affinity of Mmi1 for RNA positively correlates with Mmi1-mediated acceleration of deadenylation by Ccr4-Not: mutation of the central As (*rec8*^UUACAC^ or *rec8*^UUCUCC^) reduces the ability of Mmi1 to stimulate deadenylation (Figures 6D and S6E).

Mutation of the central As disrupts the hairpin loop in the upstream region (Figure S3C), increasing the single-stranded nature of the substrate. This increases the rate of deadenylation by Ccr4-Not without Mmi1, compared to the wild-type DSR. The small Mmi1 stimulation on this RNA may be similar to the effect of Mmi1 on the deadenylation of the 20-mer-A_30_ substrate (Figures S5B and S5D) and may be due to the non-specific RNA-binding activity of Mmi1 (Figure S7B). Thus, these results suggest that the rate of deadenylation by Mmi-Ccr4-Not correlates both with affinity of the YTH-RNA interaction and with the sequence/structure of the RNA.

### A Low-Complexity Region in Mmi1 Is Required for Interaction with Ccr4-Not and Stimulation of Deadenylation Activity

**Figure 7:**
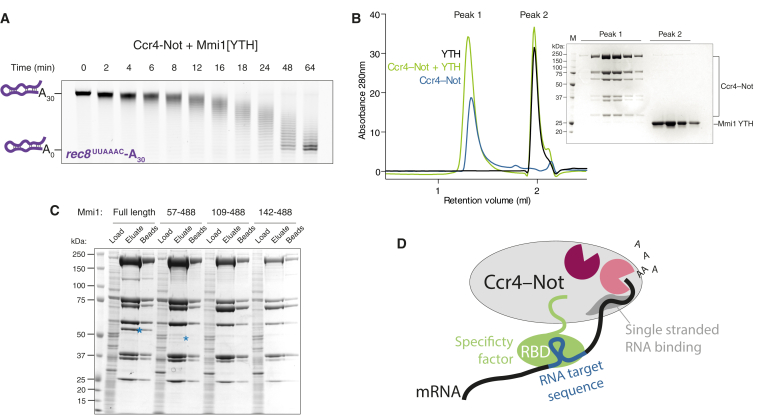
An N-terminal Low-Complexity Region in Mmi1 Is Critical for Stable Interaction with Ccr4-Not and Stimulation of Deadenylation (A) The Mmi1 YTH domain does not stimulate deadenylation in *trans*. Deadenylation assay was performed on the *rec8*-A_30_ substrate with recombinant Ccr4-Not and an equimolar quantity of Mmi1 YTH RNA-binding domain. (B) Analytical size exclusion chromatogram with SDS-PAGE of peak fractions demonstrating that Ccr4-Not and the Mmi1 YTH domain construct do not co-migrate. Ccr4-Not alone (blue) and the Mmi1 YTH domain alone (black) are also shown. M, molecular weight marker. (C) The first 56 N-terminal amino acids of Mmi1 are critical for stable complex formation with Ccr4-Not, as shown in SDS-PAGE analysis of pull-downs of proteins expressed from recombinant baculoviruses containing Mmi1 N-terminal deletions. Blue asterisks denote the Mmi1 protein where it can be observed. (D) Model for targeted deadenylation by Ccr4-Not. The low-complexity region of a specificity factor (green, e.g., Mmi1) is required for interaction with Ccr4-Not (gray, shown with two deadenylases in pink), whereas the RNA-binding domain of a specificity factor (e.g., the C-terminal YTH domain of Mmi1) binds target mRNAs (e.g., DSR sequence, blue), resulting in mRNA deadenylation. The Ccr4-Not complex may also contain an intrinsic single-stranded-RNA-binding activity.

The YTH domain of Mmi1 is preceded by an ∼35-kDa amino-terminal low-complexity (serine-rich) region (Figure S7A). Since the YTH domain of Mmi1 is sufficient for RNA binding, we tested whether it is also sufficient to accelerate deadenylation by Ccr4-Not. Addition of an equimolar amount of the purified Mmi1 YTH domain to Ccr4-Not did not stimulate the rate of deadenylation (Figure 7A; compare with Figure 4B). This suggests that the low-complexity N-terminal region is required for Mmi1 activity.

To determine whether the YTH domain binds to Ccr4-Not directly, we performed analytical size exclusion chromatography. Ccr4-Not and the YTH domain did not co-elute (Figure 7B). Thus, our data are consistent with a role for the N-terminal low-complexity region of Mmi1 in binding Ccr4-Not. This would tether the C-terminal RNA-binding YTH domain to the complex and would explain why the YTH domain alone is not able to stimulate deadenylation activity. Instead, addition of excess YTH domain inhibits Mmi1-Ccr4-Not, likely by preventing Mmi1-DSR interactions (Figures S5C and S5D).

Finally, we used truncation mutants of Mmi1 to determine which part of the protein is essential for interaction with Ccr4-Not. We co-expressed these truncations with Ccr4-Not in *Sf*9 cells. By purifying the complex using a StrepII tag on Caf1, we could evaluate whether the Mmi1 variants still interact with the complex. Deletion of 56 N-terminal amino acids was sufficient to eliminate binding (Figure 7C). Thus, the extreme N terminus of Mmi1 is required for interaction with Ccr4-Not.

## Discussion

Here, we describe a fully recombinant Ccr4-Not complex that acts as an active deadenylase in vitro. Moreover, we reconstitute targeted deadenylation using the RNA-binding protein Mmi1 as a model specificity factor. Our results suggest that specificity factors tether RNAs to Ccr4-Not to accelerate their deadenylation.

### A Fully Recombinant Deadenylation System

Poly(A) tail length control is a dynamic process that is regulated by multiple factors. Many associated binding proteins interact with Ccr4-Not, often via the Not1 scaffold protein. Our fully recombinant system will facilitate dissection of the effects of these various factors on complex activity.

Previous work characterized the activities of the individual nuclease subunits. This showed that both Caf1 and Ccr4 are ribonucleases highly specific for adenosine (Andersen et al., 2009, Wang et al., 2010). Our data with the intact complex also shows high specificity for the 30-mer poly(A) tail of our substrates. However, the entire complex demonstrates more robust activity than the nuclease module alone. We note that, in some previous work, the isolated enzymes show relatively low activities with a 10- to 100-fold molar excess required for efficient deadenylation (Jonstrup et al., 2007, Wang et al., 2010). Increased activity in the context of the intact complex could be due to allosteric activation or stabilization of the nucleases, or an RNA-binding surface contributed by other complex components. Interestingly, both Caf1 and Ccr4 are active in our intact complex—it remains unclear whether they have alternative functions in vivo or in specific situations.

Interestingly, even in the absence of an RNA adapter, our in vitro system shows that the deadenylation activity of Ccr4-Not is influenced by the RNA sequence upstream of the poly(A) tail. Thus, Ccr4-Not may have an intrinsic sequence preference, or an RNA secondary structure may influence its activity. The *rec8*^UUAAAC^-A_30_ substrate has the propensity to adopt a stem-loop structure (Figure S3) and is deadenylated more slowly than an unstructured substrate. Furthermore, our experiments with complimentary oligonucleotides annealed to the non-A portion of the RNA substrate, and a substrate with a U-rich upstream sequence, demonstrate that a stable duplex substantially inhibits deadenylation activity. These properties could be due to a single-stranded-nucleic-acid-binding capacity intrinsic to the complex (Figure 7D). A structure of the yeast NOT module (portions of Not1, Not2, and Not5) revealed a potential RNA-binding surface with poly(U) specificity (Bhaskar et al., 2013). Other Ccr4-Not subunits may thus confer binding activity to the poly(A) tail or to the upstream 3′ UTR. Also, in agreement with this, mRNA isoforms with poly(U) tracts or 3′ stem-loop structures are stabilized in yeast (Geisberg et al., 2014). This could be due to Ccr4-Not’s intrinsic RNA preference, but further experiments will be required to understand the molecular basis of sequence dependence on deadenylation activity.

An advantage of utilizing the entire Ccr4-Not complex is the ability to assay activity with RNA-binding protein adapters. Previously, it was shown that a partially purified native Ccr4-Not complex from *S. cerevisiae* is recruited to specific RNAs by Puf proteins (Goldstrohm et al., 2006, Hook et al., 2007). Similarly to Mmi1, this showed an acceleration of deadenylation. However endogenous complex preparations yield low amounts of material, contain substoichiometric components, and are contaminated with other host protein factors. Our recombinant preparation allows assays to be conducted with a complex containing pure components in unit stoichiometry with strict control of the accessory protein composition.

### Mmi1 as a Model Specificity Factor

Mmi1 regulates meiosis by binding to specific RNAs. Interestingly, CRAC (UV cross-linking and analysis of cDNA) and RNA sequencing (RNA-seq) experiments show that Mmi1 regulates expression of many mRNAs and non-coding RNAs (Kilchert et al., 2015). This indicates that Mmi1 plays a more general role in RNA repression and is not limited to meiotic transcripts. Still, the molecular mechanisms governing Mmi1-mediated repression are not well understood.

We show that Mmi1 interacts stably with native and recombinant Ccr4-Not. Thus, one method by which Mmi1 could facilitate repression of gene expression is through targeting mRNAs for deadenylation. By binding Ccr4-Not in a manner dependent on the N-terminal low-complexity region, Mmi1 likely tethers DSR-containing RNAs that are bound to its C-terminal YTH domain (Figure 7D). We show that this promotes deadenylation of target RNAs by Ccr4-Not in vitro, and there is a correlation between this activity and the affinity of the YTH domain for RNA.

Interestingly, many of the RNAs that co-purify with Mmi1 in RNA immunoprecipitation experiments also co-purify with the Ccr4-Not complex, suggesting a possible functional link (Cotobal et al., 2015). Importantly, recruitment of Ccr4-Not to these mRNAs is lost in *mmi1Δ* cells (Cotobal et al., 2015), in agreement with our results that Mmi1 acts as a specificity factor in vitro. Another recent study also showed that Mmi1 associates with purified native Ccr4-Not from *S. pombe* (Ukleja et al., 2016). Together, this suggests that Mmi1 could act as a specificity factor for Ccr4-Not, targeting specific mRNAs for robust deadenylation. Future work will dissect the connections between Mmi1-mediated deadenylation, heterochromatin formation, and mRNA repression.

In addition to Mmi1, recent work suggests that other YTH domain proteins may also be involved in RNA decay. For example, mammalian YTHDF2 binds m^6^A-containing mRNAs with a C-terminal YTH domain (Wang et al., 2014). A low-complexity N-terminal region likely targets the bound RNA for deadenylation by Ccr4-Not (Du et al., 2016). The *S. cerevisiae* YTH-domain protein Pho92 regulates mRNA stability of genes involved in phosphate signal transduction, including the Pho4 transcription factor (Kang et al., 2014). Pho92 co-immunoprecipitates with Caf1 (Kang et al., 2014). Thus, YTH proteins are linked with regulating gene expression across eukaryotes. By controlling expression of key transcription factors (e.g., Pho4 and Mei4), YTH proteins may act as master regulators of specific processes.

### RNA-Binding Adapters Tether Substrate RNAs to Ccr4-Not

Artificially tethering Ccr4-Not to reporter RNAs in vivo results in their deadenylation (Cooke et al., 2010, Finoux and Séraphin, 2006). Now, reconstitution of targeted deadenylation in vitro using an RNA-binding protein that interacts with purified Ccr4-Not allows further investigation of this process. We used Mmi1 as a model specificity factor because it was present in high abundance in our native Ccr4-Not preparations and stably bound to the complex. This acts a paradigm for other RNA-binding proteins that are specificity factors for Ccr4-Not (Figure 7D). Like Mmi1, other RNA-binding proteins often use low-complexity regions to bind Ccr4-Not (Bhandari et al., 2014, Chen et al., 2014, Fabian et al., 2013, Mathys et al., 2014, Raisch et al., 2016). We suggest that at least three factors contribute to the diversity in stabilities of different mRNAs in cells (Presnyak et al., 2015): affinity of an RNA-binding protein for Ccr4-Not, affinity of the RNA-binding protein for RNA, and sequence context (single-stranded nature) of the mRNA.

To understand the molecular mechanism of Ccr4-Not specificity, we will need to investigate how low-complexity regions tether RNA-binding domains to the complex. This will be challenging because other specificity factors may interact with Ccr4-Not more transiently: no other RNA-binding proteins co-purified with native Ccr4-Not in large quantity. Extensive low-complexity sequences often cause purified proteins to be aggregated, insoluble, and degraded, making in vitro reconstitutions difficult. Future work will address these problems using our fully recombinant in vitro system to produce complexes containing other specificity factors and to test whether Ccr4-Not complexes can be functionalized with multiple RNA-binding proteins. This will allow us to understand the modularity of RNA-binding adapters on Ccr4-Not and to explain how it controls gene expression.

## Experimental Procedures

### Endogenous Ccr4-Not Purification

The *rcd1*-TAP *S. pombe* strain was a kind gift from Cristina Cotobal and Juan Mata (University of Cambridge). The Caf40-TAPS *S. cerevisiae* strain was made in house. Ccr4-Not was purified according to modified TAP protocols detailed in the Supplemental Experimental Procedures.

### Recombinant Ccr4-Not Expression

Cloning and baculovirus preparation are detailed in the Supplemental Experimental Procedures. *Sf*9 pre-cultures were infected with virus stock and maintained for 72 hr. These infected cells were used to inoculate *Sf*9 suspension cultures (0.5 L at 2 × 10^6^ cells per milliliter in 2-L flasks) at 1:100 dilution. Expression cultures were incubated at 140 rpm and 27°C for a further 48–60 hr. Cells were harvested by centrifugation at 2,200 × *g* for 10 min and washed in ice-cold PBS, and pellets were flash frozen in liquid nitrogen and stored at −80°C.

### Recombinant Ccr4-Not Purification

Frozen *Sf*9 pellets from 4 L culture were thawed in lysis buffer: 100 mM HEPES (pH 8.0), 300 mM NaCl, 2 mM Mg(OAc)_2_, 1 mM CaCl_2_, 2 mM DTT, 5% (w/v) glycerol, protease inhibitor cocktail (Roche), 0.4 mM PMSF, and DNase I (5 μg/ml) (Sigma). Cells were lysed using sonication, and lysate was cleared using ultracentrifugation at 100,000 × *g* for 20 min. Clarified lysate was bound in batch to 5 mL Strep-Tactin Superflow Resin (IBA Lifesciences) for 2 hr. Beads were washed with 50 mM HEPES (pH 8.0), 150 mM NaCl, 2 mM Mg(OAc)_2_, and 2 mM DTT before elution in buffer supplemented with 5 mM desthiobiotin (IBA Lifesciences). Eluate was loaded onto a HiTrap Q HP 5-mL column (GE Healthcare), and the protein was eluted using a 12-column-volume gradient. Peak fractions were collected and pooled. The tag was cleaved overnight at 4°C using 0.25–0.5 mg TEV (tobacco etch virus) protease (produced in house). Ccr4-Not was then subjected to size exclusion chromatography using a Superose 6 Prep Grade XK16/70 column (GE Healthcare) equilibrated with 20 mM HEPES (pH 8.0), 150 mM NaCl, 2 mM Mg(OAc)_2_, and 0.5 mM TCEP. Peak fractions were pooled and loaded onto a Resource Q 1 mL column and eluted using a step gradient in 20 mM HEPES (pH 7.5), 500 mM NaCl, 2 mM Mg(OAc)_2_, and 0.5 mM TCEP. This concentrated the complex to 2–5 mg/mL, which was then flash frozen in liquid nitrogen for storage at −80°C.

### Deadenylation Activity Assays

RNAs with indicated sequences included a 3′ tail of 30 adenosines and a 5′ 6-FAM fluorophore label (Integrated DNA Technologies; IDT). Annealed duplex substrates were generated by slowly cooling samples heated to 95°C in the presence of the complementary single-stranded DNA (ssDNA) oligonucleotides in 10 mM Tris (pH 7.8) and 50 mM NaCl. Deadenylation assays were performed in 20 mM PIPES (pH 6.8), 10 mM KCl, 50 mM NaCl (from the Ccr4-Not buffer), 2 mM Mg(OAc)_2_, and 0.1 mM TCEP at 22°C. The complex activity was salt and pH sensitive, so carry-over from the added complex was carefully controlled. Complex stocks at 1 μM (10×) were made at 500 mM NaCl, so that assays contained 50 mM final NaCl concentration. Reactions containing 200 nM RNA were started by the addition of Ccr4-Not or Mmi1-Ccr4-Not complex at a final concentration of 100 nM. Samples of 4 μL were taken at the desired time points and mixed with denaturing formamide loading buffer. Samples were run on a denaturing polyacrylamide gel (20% [w/v] 19:1 acrylamide:bisacrylamide, 7 M urea, 1× TBE [Tris-borate-EDTA]) for 45 min at 400 V and imaged on a Typhoon FLA-7000 laser scanner (GE Healthcare).

### Fluorescence Polarization

Mmi1 was purified as detailed in Supplemental Experimental Procedures. Proteins were incubated at room temperature for 10 min with 10 nM 3′-6FAM-labeled *rec8* RNAs (sequences are as indicated in the figures; synthesized by IDT and Dharmacon) in 20 mM HEPES (pH 7.5), 100 mM NaCl, 1 mM Mg(OAc)_2_, and 0.1 mM TCEP. Fluorescence polarization was measured using a PHERAstar *Plus* microplate reader (BMG Labtech). Dissociation constants were estimated by Hill-slope non-linear regression in GraphPad Prism 6. Error bars indicate the SD of five biological replicates (each with three technical replicates).

## Author Contributions

J.A.W.S. performed or contributed to all experiments. M.W.W. designed and performed some of the biochemical assays. J.W. and K.L.S. purified native complexes from yeast. A.K. made some baculoviruses and performed protein purifications in the early stages of the project. L.A.P. conceived and supervised the project. J.A.W.S., M.W.W., and L.A.P. wrote the manuscript.

## Supplemental Information

Document S1. Supplemental Experimental Procedures and Figures S1–S7

Document S2. Article plus Supplemental Information
