# Supplementary material for: Reconstitution of Targeted Deadenylation by the Ccr4-Not Complex and the YTH Domain Protein Mmi1
Source: Cell Rep. 2016 Nov 15;17(8):1978–89. doi: 10.1016/j.celrep.2016.10.066 (PMC5120349; doi:10.1016/j.celrep.2016.10.066)
Supplement: Document S1. Supplemental Experimental Procedures and Figures S1–S7 [file mmc1.pdf]

Cell Reports, Volume *17*

Supplemental Information

Reconstitution of Targeted Deadenylation

by the Ccr4-Not Complex and the YTH

Domain Protein Mmi1

James A.W. Stowell, Michael W. Webster, Alexander Kögel, Jana Wolf, Kathryn L. Shelley, and Lori A. Passmore

```
Sc_Ccr4       1 MNDPSLLGYPNVGPQQQQQQQQQQHAGLLGKGTPNALQQQLHMNQLTGIPPPGLMNNSDVHTSSNNNSRQLLDQLANGNA 80
Sp_Ccr4         --------------------------------------------------------------------------------
Hs_CNOT6        --------------------------------------------------------------------------------
Hs_CNOT6L       --------------------------------------------------------------------------------

Sc_Ccr4      81 NMLNMNMDNNNNNNNNNNNNNNNGGGSGVMMNASTAAVNSIGMVPTVGTPVNINVNASNPLLHPHLDDPSLLN------- 153
Sp_Ccr4       1 -----------------------------MFNPR-------------YT-----QGTIYPGAHPGLLTPDHQHAAILSVQ 33
Hs_CNOT6        --------------------------------------------------------------------------------
Hs_CNOT6L       --------------------------------------------------------------------------------

Sc_Ccr4     154 ----------NPIWKLQLHLAAVSAQSLGQPNIYARQNAMKKYLATQQAQQAQQQAQQQAQQQVPGPFGPGPQAAPPALQ 223
Sp_Ccr4      34 NSPALENSNISEHWKQQIALATQSR-SFSSPHQ---------------------RAHNA--AALARSGGPGFSMN----- 84
Hs_CNOT6        --------------------------------------------------------------------------------
Hs_CNOT6L       --------------------------------------------------------------------------------

Sc_Ccr4     224 PTDFQQSHIAEASKSLVDCTKQALMEMADTLTDSKTAKKQQPTGDSTPSGTATNSAVSTPLTPKIELFANGKDEANQALL 303
Sp_Ccr4      85 ---Y-------------NARTGAFTGGPNAA------GLSSLGGKYNTSSTTTTLTTSTTLNT------SSGT----TLN 132
Hs_CNOT6      1 --------------------------M--------------PKEKYEPPDPRR--------------------------- 13
Hs_CNOT6L     1 ---------------------MRLIGM--------------PKEKYDPPDPRR--------------------------- 18

Sc_Ccr4     304 QHKKLSQYSIDEDDDIENRMVMPKDSKYDDQLWHALDLSNLQIFNISANIFKYDFLTRLYLNGNSLTELPAEIKNLSNLR 383
Sp_Ccr4     133 STSKT-----TTSSVAVDDQKSKSDSKKERRDWTCLDLGGIGLRNVSTDLFKFSFLTELYINHNNLTRLPPEIGKLKNLV 207
Hs_CNOT6     14 ---------------MYTIMSSEEAANGKKSHWAELEISG-KVRSLSASLWSLTHLTALHLSDNSLSRIPSDIAKLHNLV 77
Hs_CNOT6L    19 ---------------IYTIMSAEEVANGKKSHWAELEISG-RVRSLSTSLWSLTHLTALHLNDNYLSRIPPDIAKLHNLV 82

Sc_Ccr4     384 VLDLSHNRLTSLPAELGSCFQLKYFYFFDNMVTTLPWEFGNLCNLQFLGVEGNPLEKQFLKILTE-KSVTGLIFYLRDNR 462
Sp_Ccr4     208 ILDASGNSIKTIPPELGLLTELREVLLFDNMISVIPAELGTLFQLKILGIEGNPLQDVYKNQIME-SGTAGLIAALRDGC 286
Hs_CNOT6     78 YLDLSSNKIRSLPAELGNMVSLRELHLNNNLLRVLPFELGKLFQLQTLGLKGNPLTQDILNLYQEPDGTRRLLNYLLDNL 157
Hs_CNOT6L    83 YLDLSSNKLRSLPAELGNMVSLRELLLNNNLLRVLPYELGRLFQLQTLGLKGNPLSQDILNLYQDPDGTRKLLNFMLDNL 162

Sc_Ccr4     463 P------EIPLPHERRFIEINTDGEPQREYDS-------LQQSTEHLATDLAKRTFTVLSYNTLCQHYATPKMYRYTPSW 529
Sp_Ccr4     287 P------VGPPPPERGWEKLVSDDDDDVNDNVSTSVRDLTAADSNKPSTTSKNLKFTIMSYNVLCERYATSTLYGYTPSW 360
Hs_CNOT6    158 SGTAKRITTEQPPPRSWIMLQEP------------------------DRTRPTALFSVMCYNVLCDKYATRQLYGYCPSW 213
Hs_CNOT6L   163 AV-----HPEQLPPRPWITLKER------------------------DQILPSASFTVMCYNVLCDKYATRQLYGYCPSW 213

Sc_Ccr4     530 ALSWDYRRNKLKEQILSYDSDLLCLQEVESKTFEEYWVPLLDKHGYTGIFHAKARAKTMHSKDSKKVDGCCIFFKRDQFK 609
Sp_Ccr4     361 ALSWSYRKDLIMQELGGYNADIICLQEVDVENYDTFFAPQMSLKGYKGVHFPKSRVRTMNEVERRIVDGCATFFKTSKYV 440
Hs_CNOT6    214 ALNWDYRKKAIIQEILSCNADIVSLQEVETEQYYSFFLVELKERGYNGFFSPKSRARTMSEQERKHVDGCAIFFKTEKFT 293
Hs_CNOT6L   214 ALNWEYRKKGIMEEIVNCDADIISLQEVETEQYFTLFLPALKERGYDGFFSPKSRAKIMSEQERKHVDGCAIFFKTEKFT 293

Sc_Ccr4     610 LITKDAMDFSGAWMKH-KKFQRTEDYLNRAMNKDNVALFLKLQHIP--------------SGDTIWAVTTHLHWDPKFND 674
Sp_Ccr4     441 MHEKMVIEYNQAPSLRRQDIKLTSNMYNRVMTKDNISVITLLENKEN--------------GSRLIVANCHIHWDPQFRD 506
Hs_CNOT6    294 LVQKHTVEFNQLAMANS---EGSEAMLNRVMTKDNIGVAVLLELRKESIEMPSGKPHLGTEKQLILVANAHMHWDPEYSD 370
Hs_CNOT6L   294 LVQKHTVEFNQVAMANS---DGSEAMLNRVMTKDNIGVAVVLEVHKELFGA-GMKPIHAADKQLLIVANAHMHWDPEYSD 369

Sc_Ccr4     675 VKTFQVGVLLDHLETLLKEETSH-------------NFRQDIKKFPVLICGDFNSYINSAVYELINTGRVQIHQEG-NGR 740
Sp_Ccr4     507 VKVIQVAMLMDEIAQVATKFRNMPSKIPSDQLKDERPTYPEYLKIPILICGDFNSVQGSGVYDFLSSGSISQNHEDFMNN 586
Hs_CNOT6    371 VKLVQTMMFLSEVKNIIDKASRNLKS----------SVLGEFGTIPLVLCADLNSLPDSGVVEYLSTGGVETNHKDFKEL 440
Hs_CNOT6L   370 VKLIQTMMFVSEVKNILEKASSRPGS-----------PTADPNSIPLVLCADLNSLPDSGVVEYLSNGGVADNHKDFKEL 438

Sc_Ccr4     741 DFGY------------MSEKNFSHNLALKSSYNCIGELPFTNFTPSFTDVIDYIWFSTHALRVRGLLGEVDPEYVS--KF 806
Sp_Ccr4     587 DYGEYTVN------------GRSHAFNLKSAYGESEALSFTNYTPGFKGAIDHIWYTGNSLEVTGLLKGVDKDYLS--GV 652
Hs_CNOT6    441 RYNESLTNFSCHGKNGTTNGRITHGFKLQSAYE-SGLMPYTNYTFDFKGIIDYIFYSKPQLNTLGILGPLDHHWLVENNI 519
Hs_CNOT6L   439 RYNECLMNFSCNGKNGSSEGRITHGFQLKSAYE-NNLMPYTNYTFDFKGVIDYIFYSKTHMNVLGVLGPLDPQWLVENNI 517

Sc_Ccr4     807 IGFPNDKFPSDHIPLLARFEFMKTNTGSKKV-------                                           837
Sp_Ccr4     653 VGFPNAHFPSDHICLLAEFKVKQEKTPLPSSKFNNDKK                                           690
Hs_CNOT6    520 SGCPHPLIPSDHFSLFAQLELLLPFLPQVNGIHLPGRR                                           557
Hs_CNOT6L   518 TGCPHPHIPSDHFSLLTQLELHPPLLPLVNGVHLPNRR                                           555


Sc_Pop2       1 MQSMNVQPRVLAVGGEQFFSQRQASEQHQQQNMGPQVYSPKVNRARMFPQGMPVNTINGSVNQEMNNAYLLKQKNEPLLT 80
Sp_Caf1         --------------------------------------------------------------------------------
Hs_CNOT7        --------------------------------------------------------------------------------
Hs_CNOT8        --------------------------------------------------------------------------------

Sc_Pop2      81 QQQQQQQQQQPFNIGTPVSVASLPPGLNVLQQQQQQQQQQQQQQQQGVGLNRPLASQLPKHLTNQSMPPIFL--PPPNYL 158
Sp_Caf1       1 ---------------------------------------------------------MTAMNSNFSYPALGVDGISSQIS 23
Hs_CNOT7      1 ------------------------------------------------------------------MPAA----TVDHSQ 10
Hs_CNOT8      1 ------------------------------------------------------------------MPAA----LVENSQ 10

Sc_Pop2     159 FVRDVWKSNLYSEFAVIRQLVSQYNHVSISTEFVGTLARPIGTFRSKVDYHYQTMRANVDFLNPIQLGLSLSDANGNKPD 238
Sp_Caf1      24 PIRDVWSTNLQQEMNLIMSLIERYPVVSMDTEFPGVVARPLGVFKSSDDYHYQTLRANVDSLKIIQIGLALSDEEGNAPV 103
Hs_CNOT7     11 RICEVWACNLDEEMKKIRQVIRKYNYVAMDTEFPGVVARPIGEFRSNADYQYQLLRCNVDLLKIIQLGLTFMNEQGEYPP 90
Hs_CNOT8     11 VICEVWASNLEEEMRKIREIVLSYSYIAMDTEFPGVVVRPIGEFRSSIDYQYQLLRCNVDLLKIIQLGLTFTNEKGEYPS 90

Sc_Pop2     239 NGPSTWQFNFEFDPKKEIMSTESLELLRKSGINFEKHENLGIDVFEFSQLLMDSGLMMDDSVTWITYHAAYDLGFLINIL 318
Sp_Caf1     104 -EACTWQFNFTFNLQDDMYAPESIELLTKSGIDFKKHQEVGIEPADFAELLIGSGLVLQEEVTWITFHSGYDFAYLLKAM 182
Hs_CNOT7     91 -GTSTWQFNFKFNLTEDMYAQDSIELLTTSGIQFKKHEEEGIETQYFAELLMTSGVVLCEGVKWLSFHSGYDFGYLIKIL 169
Hs_CNOT8     91 -GINTWQFNFKFNLTEDMYSQDSIDLLANSGLQFQKHEEEGIDTLHFAELLMTSGVVLCDNVKWLSFHSGYDFGYMVKLL 169

Sc_Pop2     319 MNDSMPNNKEDFEWWVHQYMPNFYDLNLVYKIIQEFKNPQLQQSSQQQQQQQYSLTTLADELGLPRFSIFTTTGGQSLLM 398
Sp_Caf1     183 TQIPLPAEYEEFYKILCIYFPKNYDIKYIMKSVLNNSK---------------GLQDIADDLQIHRIGPQHQAGSDALLT 247
Hs_CNOT7    170 TNSNLPEEELDFFEILRLFFPVIYDVKYLMKSCKNLKG---------------GLQEVAEQLELERIGPQHQAGSDSLLT 234
Hs_CNOT8    170 TDSRLPEEEHEFFHILNLFFPSIYDVKYLMKSCKNLKG---------------GLQEVADQLDLQRIGRQHQAGSDSLLT 234

Sc_Pop2     399 LLSFCQLSKLSMHKFPNGTDFAKYQGVIYGIDGDQ--------------------------------------------- 433
Sp_Caf1     248 ARIFFEIRS---RYFDGSIDS-RMLNQLYGLGSTGSVLWHNNSSTPQIQFRDLPGAHPSPTPSNAGIPTTLTNTSSAPNF 323
Hs_CNOT7    235 GMAFFKMRE---MFFEDHIDDAKYCGHLYGLGSGSSYVQNGTGNAYEEEANKQ---------------S----------- 285
Hs_CNOT8    235 GMAFFRMKE---LFFEDSIDDAKYCGRLYGLGTGVAQKQNEDVDSAQEKMSIL---------------AIINNMQQ---- 292

Sc_Pop2         ------------
Sp_Caf1     324 ANSTFRFPPRVV 335
Hs_CNOT7        ------------
Hs_CNOT8        ------------
```

Figure S1 - Ccr4 and Caf1 protein sequences. Related to Figure 1.

Sequence alignments of Ccr4 and Caf1 from *Saccharomyces cerevisiae* (Sc), *Schizosaccharomyces pombe* (Sp) and *Homo sapiens* (Hs). Sequences are highlighted shades of purple according to BLOSUM62 scores. Glutamine- and asparagine-rich regions in *S. cerevisiae* proteins are coloured magenta. Sequence alignments were performed using Clustal Omega and formatted using JalView (Waterhouse et al., 2009).

A

Protein	Species origin	Exclusive spectrum count	Percent coverage
Not1	*S. pombe*	747	82
Not3	*S. pombe*	158	73
Ccr4	*S. pombe*	125	83
Mot2/Not4	*S. pombe*	105	43
Mmi1	*S. pombe*	82	77
Not2	*S. pombe*	81	81
Rcd1	*S. pombe*	74	76
Caf1	*S. pombe*	60	62
Hsp70	*Spodoptera (Sf9)*	18	32
Beta-tubulin	*Spodoptera (Sf9)*	13	45
Alpha-tubulin	*Spodoptera (Sf9)*	10	27
Hsp90	*Spodoptera (Sf9)*	6	9
Acetyl-CoA-carboxylase	*Spodoptera (Sf9)*	6	4
E25 envelope protein	*Nuclear polyhedrosis virus*	5	29
DDX3 (predicted by homology)	*Spodoptera (Sf9)*	5	12

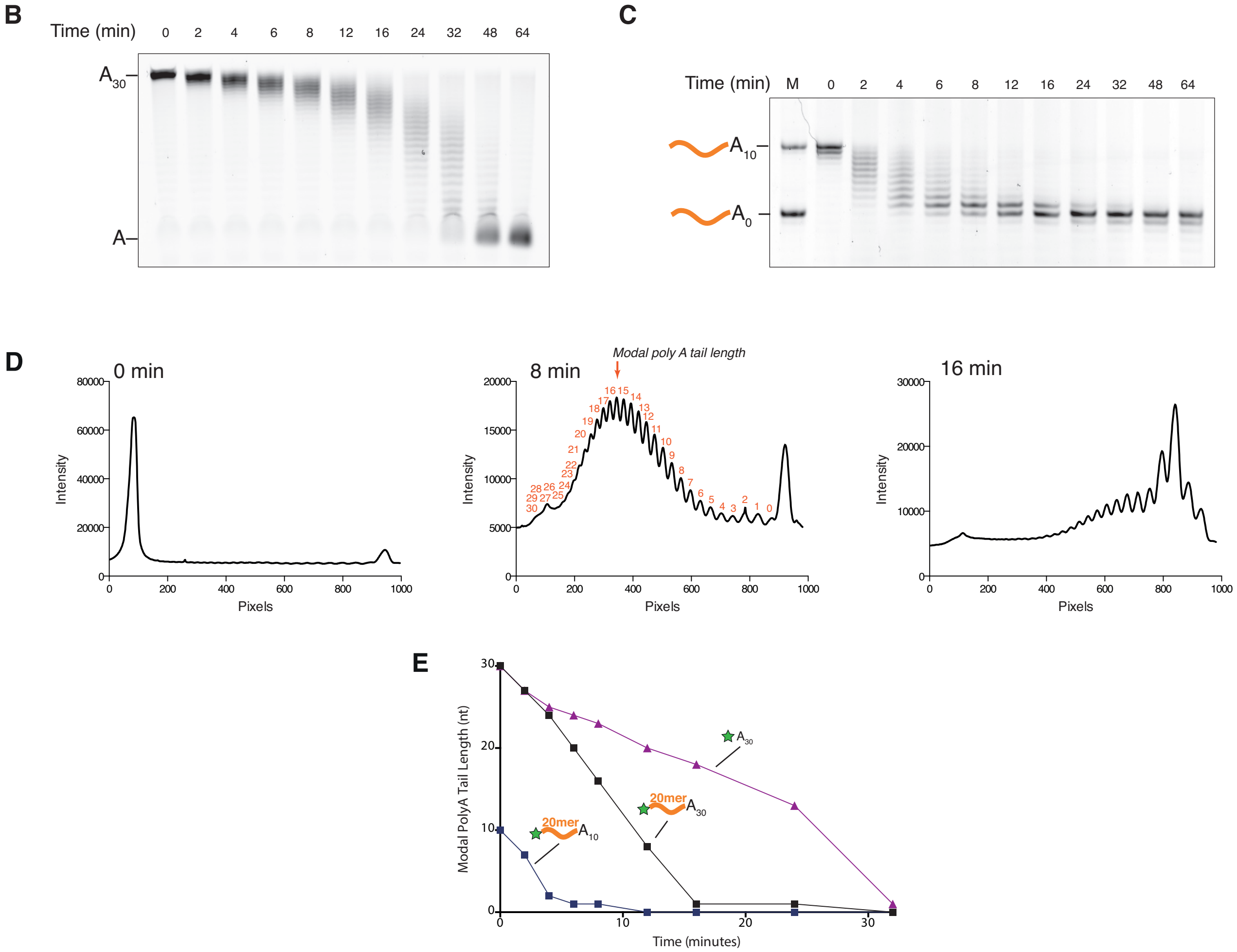

Figure S2. Generation of an active recombinant Ccr4–Not complex. Related to Figures 2 and 3.
A Table of proteins identified by LC-MS/MS of purified recombinant Mmi1-Ccr4-Not complex, ranked by exclusive spectrum count (total number of spectra uniquely assigned to this protein). The percent coverage of each protein is also shown.
B-C Deadenylation activity of recombinant Ccr4-Not, measured using (**B**) an A_{30} substrate with no upstream region and (**C**) a 20-mer–A_{10} substrate . Reactions were set up and analyzed as in Figure 3.
D-E Quantitation of deadenylation using densitometry. (**D**) Representative intensity profiles of individual gel lanes measured using ImageJ for deadenylation of the 20-mer–A_{30} RNA substrate by Ccr4-Not (from Figure S5B). Annotations on the plot for 8 minutes show how single nucleotides can be resolved in the gels so that the modal polyA tail length in a 30-mer tail can be calculated. (**E**) These modal values were taken at each time point and plotted for Ccr4-Not with 20-mer–A_{30}, 20-mer–$A_{10}$and A_{30} (see Figures 3 and S2B-C for gels).

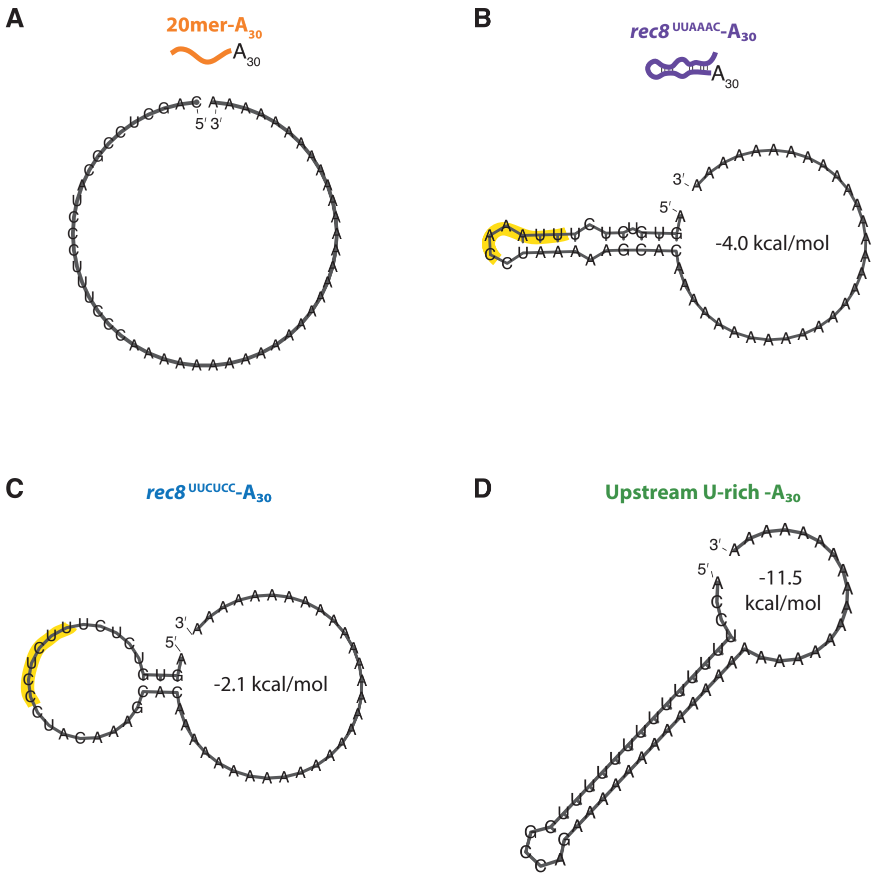

Figure S3. Predicted secondary structures of deadenylation substrates. Related to Figures 3, 4, 5 and 6.

Substrates are shown with 30-mer polyA tails as predicted structures from RNAfold (Lorenz et al., 2011), along with associated change in free-energy. (**A**) 20-mer–A_{30} was previously designed to have no secondary structure (Jonstrup et al., 2007). (**B**) *rec8* ^{UUAAAC}–A_{30}, taken from a DSR sequence within the 3′-UTR of this transcript, adopts a relatively stable stem loop structure. This stem-loop is disrupted in (**C**) *rec8* ^{UUCUCC}–A_{30} where four adenosines in the DSR and downstream region are mutated to pyrimidines. This disruption of secondary structure could explain the differences in intrinsic deadenylation rates between substrates (compare Figure 4B with Figure 6D). Wild-type and mutated DSR sequences are highlighted in yellow. (**D**) The substrate with an upstream U-rich region has the propensity to form a stem-loop with the polyA tail.

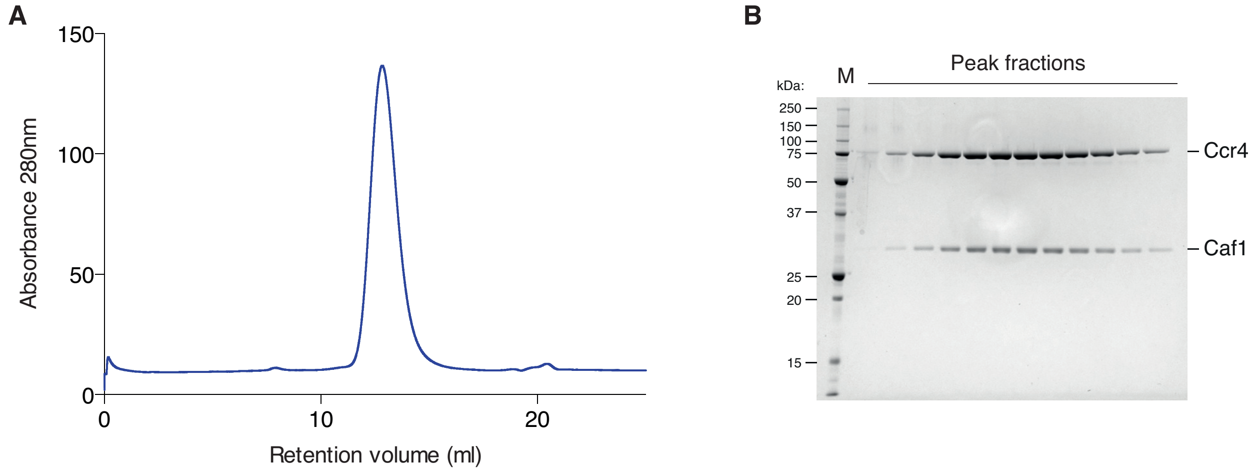

Figure S4. The Caf1–Ccr4 nuclease module forms a stable heterodimer. Related to Figure 3.
A Chromatogram of size exclusion chromatography of a recombinant Caf1–Ccr4 complex demonstrating co-elution on a Superdex 200 column.
B SDS-PAGE analysis of peak fractions from the chromatogram shown in **A.** M is molecular weight marker.

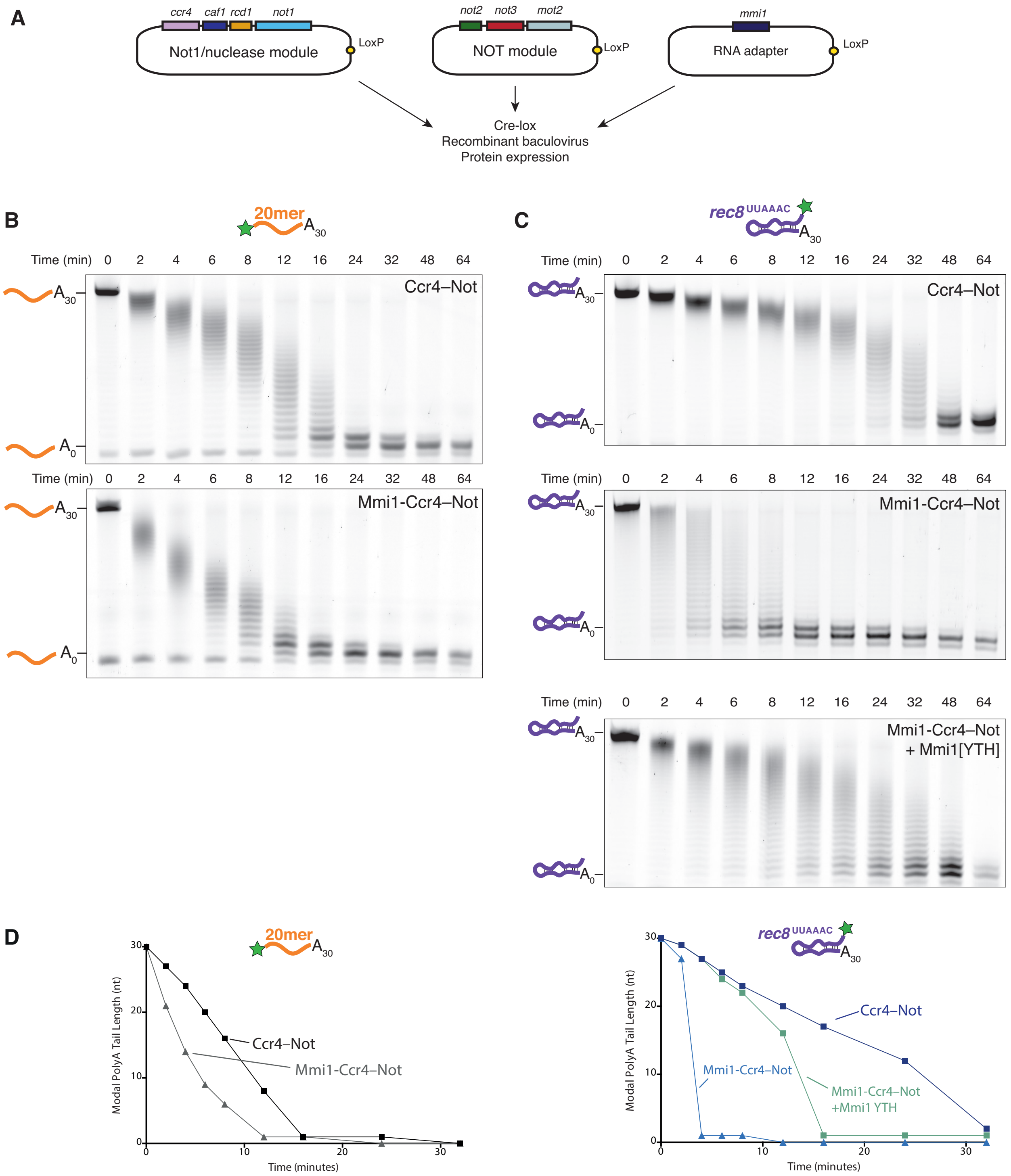

Figure S5. Generation of an active recombinant Ccr4–Not complex bound to Mmi1. Related to Figure 4.

A Scheme for the cloning and expression of a recombinant Ccr4-Not complex with Mmi1. We generated a new baculovirus transfer vector carrying the Mmi1 coding sequence. This was combined with the fully assembled Ccr4-Not vector using Cre-Lox recombination and was used to generate a single recombinant virus expressing all eight genes, as in Figure 2.

B–C Deadenylation of RNA substrates (**B** 20-mer–A_{30}; **C** *rec8*^{UUAAAC}–A_{30}) by recombinant *S. pombe* Ccr4-Not complex with and without Mmi1 was analyzed by denaturing polyacrylamide gel electrophoresis. The sizes of the RNA substrates with and without the polyA tail are shown. In **C** the bottom gel demonstrates inhibition of Mmi1-Ccr4-Not when the reaction is performed in the presence of 1 µM Mmi1 YTH RNA-binding domain added in *trans*. Some of the gels are reproduced from Figure 4.

D Plots showing modal polyA tail length as a function of time, calculated using densitometry analysis as described in Figure S2D. Plots are for 20-mer–A_{30} (left) or *rec8*^{UUAAAC}–A_{30} (right) substrates with Ccr4-Not, Mmi1-Ccr4-Not or Ccr4-Not with the Mmi1 YTH domain, as indicated.

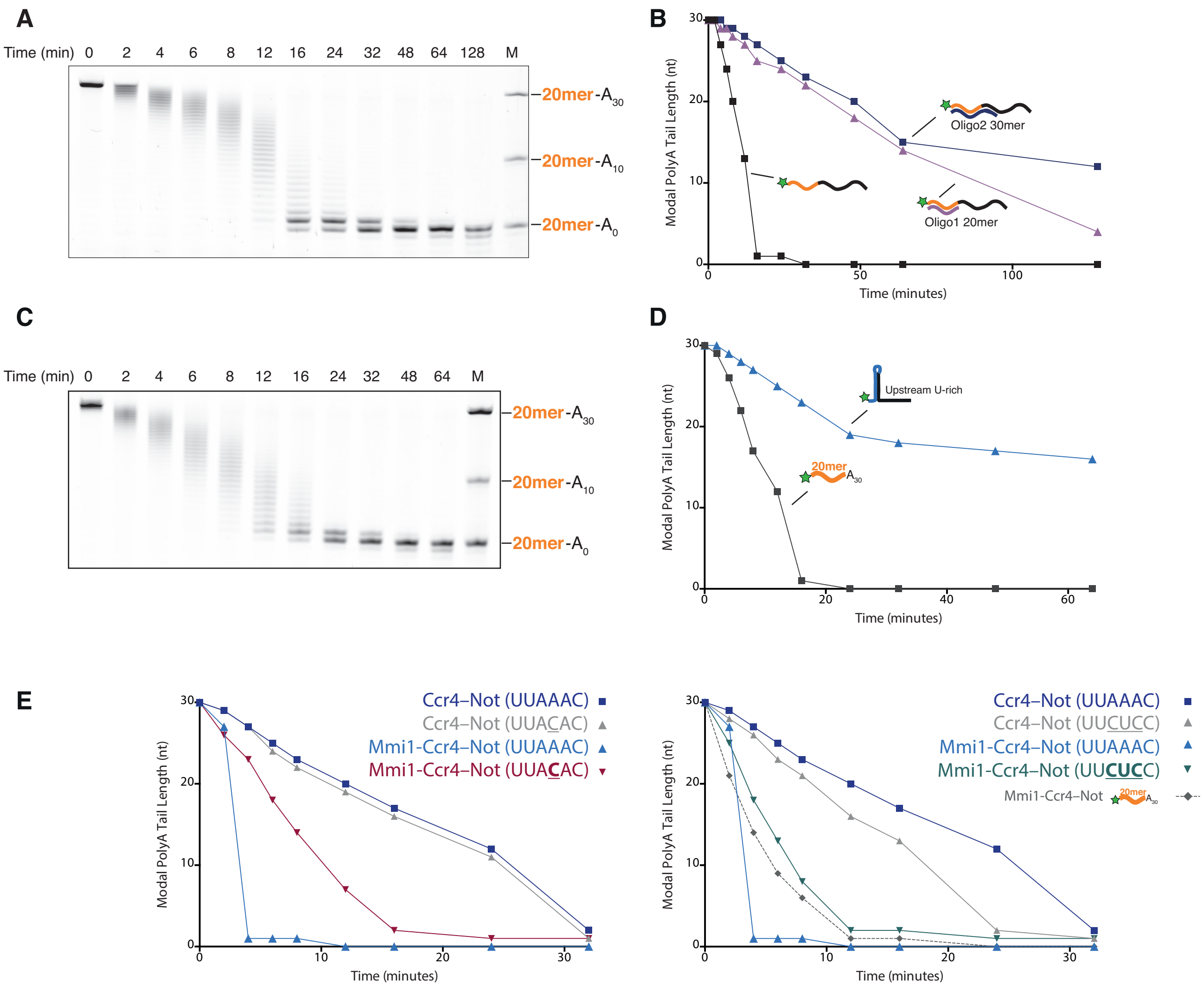

Figure S6. Quantitation of deadenylation assays. Related to Figures 5 and 6.

A–D (A,C) Control deadenylation assays on 20-mer–A_{30} performed at the same time and in exactly the same reaction conditions as Figure 5A–C (**A**) and 5D (**C**). **(B,D)** Plots showing deadenylation rates of Ccr4-Not on substrates with primers annealed to the 5′ regions of the substrates (**B**) or with an upstream uridine tract (**D**). Plots are made the same way as in S2D and are from gels shown in Figure 5.

E Plots showing deadenylation rates of *rec8* RNAs containing mutated DSR motifs with Mmi1-Ccr4–Not complexes from Figure 6D. Ccr4-Not (dark blue) and Mmi1-Ccr4-Not (light blue) complexes on wild-type *rec8*^{UUAAAC}–A_{30} substrates are shown for comparison. Plots are made the same way as Figure S2D.

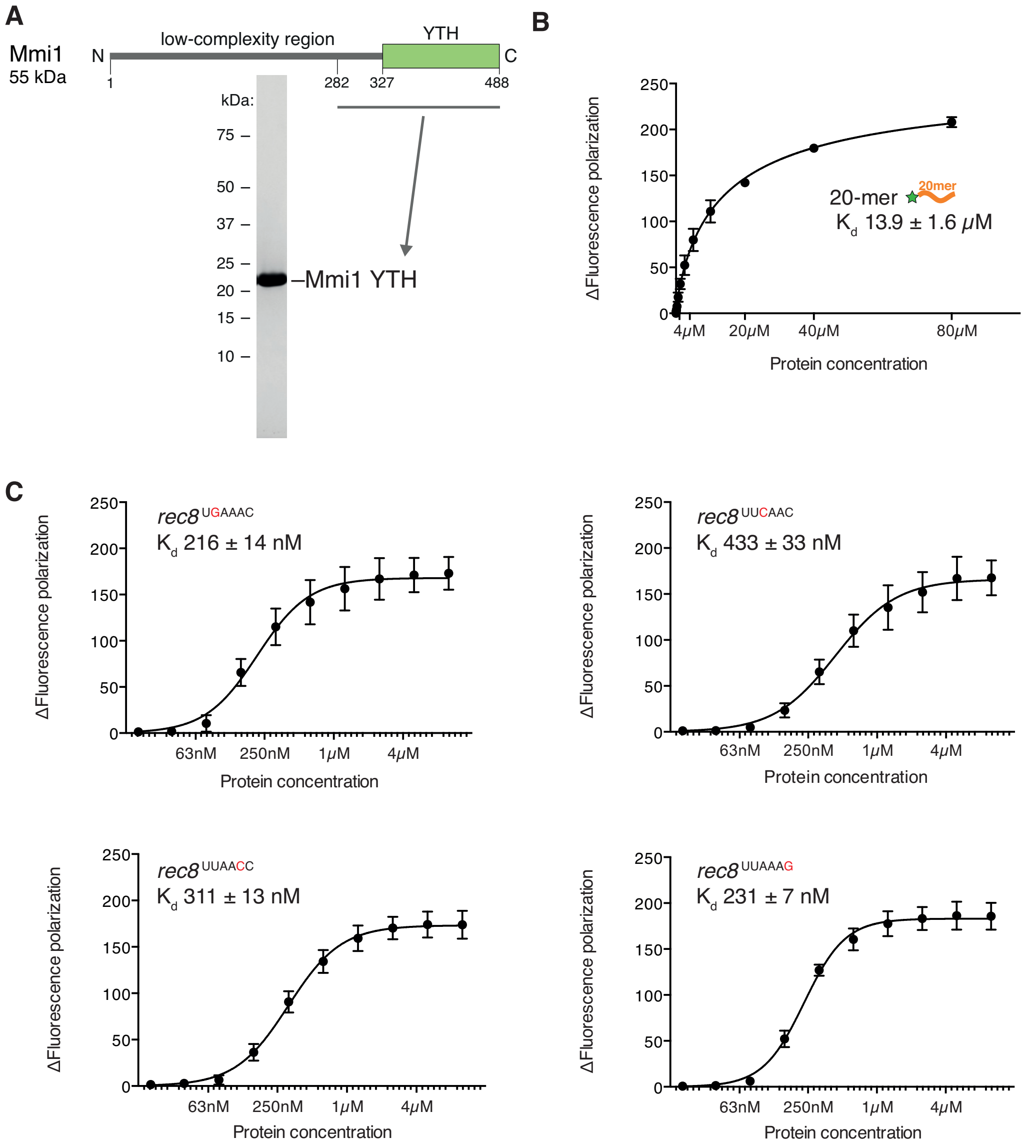

Figure S7. Specificity of Mmi1 YTH – DSR interaction. Related to Figure 6.

A Schematic diagram of full-length Mmi1 and SDS-PAGE analysis of the purified Mmi1 YTH domain construct used in all assays.

B Fluorescence polarization assay of the Mmi1 YTH-domain binding to the 20-mer unstructured upstream region (no polyA tail). Increasing concentrations of protein were added to the fluorescently labelled RNA. The mean of five dilution series replicates is plotted with error bars showing standard deviation. Data were fitted with a Hill-slope, with the calculated K_d shown.

C Fluorescence polarization assays of Mmi1 YTH binding to RNAs containing substituted DSR motifs. Two-fold dilution series of purified protein were added to fluorescently-labeled *rec8* RNAs as shown in Figure 6A but with the substitutions shown. Data were acquired, plotted and K_ds calculated as in **B**.

Supplemental Experimental Procedures

Endogenous *S. cerevisiae* Ccr4–Not purification

Ccr4–Not was purified from an *S. cerevisiae* strain where the *CAF40* gene is modified to encode a C-terminal TAPS tag (StrepII-TEV-IgG binding domain) (Schreieck et al., 2014) with genotype *CAF40-TAPS::kanMX6 MATalpha pra1-1 prb1- 1 prc1-1 cps1-3 ura3delta5 leu2-3 his-*. A single colony was used to inoculate 25 ml YPD starter culture, which was incubated for 24 h at 30°C, 200 rpm. 24 l YPD culture was inoculated with starter culture to reach a final $OD_{600\ nm}$ of 5.0-5.5 after growth for 16 h at 30°C, 200 rpm. Cells (~260 g) were harvested at 6700 *g* for 20 min, 4°C. Pellets were washed in MilliQ water, flash frozen in liquid nitrogen and stored at -80°C.

For purification, the cells were thawed, resuspended 1:1 (w/v) in buffer A (100 mM HEPES pH 7.9, 300 mM NaCl, 10% (w/v) glycerol, 2 mM DTT, complete EDTA-free protease inhibitor tablets (Roche) and 0.1 µg/ml DNaseI) and lysed at 35 kpsi in a C Series Cell Disruptor (Constant Systems) chilled to 4°C. The lysate was clarified at 41,000 rpm for 30 min, 4°C. Clarified supernatant was incubated with 4 ml bed volume IgG sepharose FF beads (GE Healthcare) and rotated for 1 h, 4 °C. Beads were washed with 100 ml buffer B (20 mM HEPES pH 7.9, 150 mM NaCl, 1 mM $Mg(OAc)_2$, 2 mM DTT), and then transferred into 4 x 2 ml tubes. The tag was cleaved with 1.2 mg TEV protease in 1.6 ml buffer B at 16°C for 1.5 hrs, 300 rpm.

Next the clarified TEV eluate was incubated with 1 ml StrepTactin Sepharose HP beads (GE Healthcare) for 45 min at 4°C. Beads were washed in 1 ml buffer B, then four times with 500 µl buffer C (20 mM HEPES pH 7.9, 400 mM NaCl, 1 mM $Mg(OAc)_2$, 2 mM DTT), two times with 500 µl buffer B and finally resuspended 1:1 (beads bed volume:buffer) in buffer D (buffer B with 5 mM desthiobiotin). Beads were incubated for 45 min at 4°C to elute the protein, then the supernatant was collected.

StrepTactin eluate samples were concentrated using an Amicon Ultra 0.5 ml 50 kDa cut-off centrifuge concentrator (Millipore) at 3000 *g*, 4°C to ~50-60 µl. The sample was loaded onto a 2.4 ml Superose 6 PC 3.2/30 column (GE Healthcare) equilibrated in buffer B. Peak fractions (1.15 – 1.4 ml) were pooled and the concentration determined.

Endogenous *S. pombe* Ccr4–Not purification

The *rcd1*-TAP strain was a kind gift from Cristina Cotobal and Juan Mata (University of Cambridge). Ccr4–Not was purified from 18 l of *S. pombe* at OD_{600nm} of 3. *S. pombe* cells were harvested by centrifugation, pellets washed with water and resuspended 1:1 (w/v) with ice cold lysis buffer (100 mM HEPES pH 8.0, 300 mM NaCl, 10 % (w/v) glycerol, 2 mM DTT, 2 mM $Mg(OAc)_2$, 1 mM $CaCl_2$ and EDTA free protease inhibitor cocktail). Resuspended cells were dripped into a liquid nitrogen bath and stored at -80°C as 'popcorn'. Cells were subsequently lysed using a freezer mill (SPEX SamplePrep), lysate cleared using ultracentrifugation and batch bound for 2 hrs to 3 ml of IgG sepharose. All purification steps were performed at 4°C unless otherwise stated. Beads were collected in an Econo-Column (Bio-Rad) and washed with 25 ml wash buffer (20 mM HEPES pH 8.0, 150 mM NaCl, 1 mM $Mg(OAc)_2$ and 2 mM DTT), followed by 50 ml wash buffer supplemented with 2 mM EGTA and finally, with the initial wash buffer again. Beads were resuspended with buffer containing RNase A (10 µg/ml final concentration) and cleaved overnight with TEV protease at a final concentration of 50 µg/ml.

The supernatant (containing the cleaved protein) was supplemented with 5 mM $CaCl_2$. Eluted proteins were then batch bound to 1 ml calmodulin sepharose (GE Healthcare) for 2 hrs. Six washes of beads were performed with 20 mM HEPES pH 8.0, 150 mM NaCl, 1 mM $Mg(OAc)_2$, 2 mM $CaCl_2$ and 2 mM DTT. Bound proteins were then eluted by rotating resin for 90 minutes at 4°C in 20 mM HEPES pH 8.0, 150 mM NaCl, 1 mM $Mg(OAc)_2$, 5 mM EGTA and 2 mM DTT. Eluate was concentrated using Amicon Ultra 0.5 ml 50 kDa cut-off centrifuge concentrator.

Mass spectrometry

Purifications from budding yeast, fission yeast or *Sf*9 cells (recombinant complexes) were analysed using NUPAGE 4-12% Bis-Tris protein gels in MOPS-SDS buffer (ThermoFisher). Gels were stained for protein using Coomassie based InstantBlue (Expedeon) reagent or G-250 Coomassie in acetic acid and methanol for mass spectrometry analysis. For band identification, excised gel slices were destained, reduced and alkylated before in-gel trypsin digestion. LC-MS/MS was performed in-house using C18 Acclaim PepMap100 trapping and separating columns and a hybrid dual pressure linear ion trap mass spectrometer using data-dependent acquisition (Orbitrap Velos, Thermo Scientific, San Jose, USA). Data were searched against a protein database (UniProt KB) using the Mascot search engine program (Matrix Science, UK). Database search parameters were set with a precursor tolerance of 5 ppm and a fragment ion mass tolerance of 0.8 Da. MS/MS data were then inspected manually using the Scaffold program (Proteome Software Inc., USA).

Recombinant Ccr4–Not cloning

Genes of Ccr4–Not subunits were synthesised with codon optimization for *E. coli* (Genscript) and inserted into Multibac transfer vectors carrying a polyhedrin (Polh) promoter and an SV40 terminator sequence (Bieniossek et al., 2008). The *caf1* and *not3* genes were synthesised with C-terminal StrepII and N-terminal His tags respectively. The sequences and vectors used are as follows: Not1 (*not1* NM_001018754.2) pACEBac1, Ccr4 (*ccr4* NM_001022893.2) pACEBac1, Caf1 (*caf1* NM_001023376.2) pACEBac1, Rcd1 (*rcd1* NM_001020415.2) pACEBac1, Not3 (*not3* NM_001020217.2) pIDC, Not2 (*not2* NM_001022816.2) pIDC and Mot2 (*mot2* NM_001018475.2) pIDC. Two expression modules were generated by combining cassettes within a pACEBac1 vector (*ccr4*, *caf1*, *rcd1*, *not1*) or a pIDC vector (*not2*, *not3*, *mot2*). To achieve this, a vector containing one (or more in subsequent rounds) subunit(s) was linearized with BstXI and the subunit expression module from another vector excised with BstXI and either I-CeuI or PI-SceI and ligated into the linearized acceptor vector. Since the BstXI site from the original vector and the I-Ceu/PI-SceI site from the insert are destroyed, this can be performed iteratively. *In vitro* Cre-Lox recombination (New England Biolabs) of the assembled vectors was performed using 400 ng of pACEBac vector and 500 ng of pIDC.

For the cloning of an eight subunit Mmi1-containing vector, Mmi1 was inserted into a pIDK vector and combined with the fully assembled pACEBac-pIDC construct by *in vitro* Cre-Lox recombination. Plasmid integrity was checked using restriction digest and sequences were verified.

Ccr4–Not baculovirus production

Fully assembled Cre-loxed vectors were transformed into chemically competent DH10EmbacY cells. Colonies that had integrated the plasmid into the baculovirus genome were picked using blue/white selection and used to inoculate 5 ml LB medium with selective antibiotics (50 µg/ml Kanamycin, 10 µg/ml Gentamycin, 35 µg/ml Chloramphenicol and in cases with a third plasmid 50 µg/ml Spectinomycin). Bacmids were purified using alkaline lysis with reagents (buffers P1, P2 and N3) from Qiagen. After neutralization and pelleting the precipitate using centrifugation, the supernatant was further centrifuged to remove any residual insoluble material. Bacmids were then precipitated using 0.5 volumes isopropanol on ice for 2 minutes, then pelleted at 20,000 *g* for 20 min at 18°C. Pellets were washed with ice-cold 70% ethanol and centrifuged at 4°C three times before being resuspended in sterile 10 mM Tris pH 8.5.

10–20 µg bacmid DNA was transfected into 6-wells of $2x10^6$ adherent *Sf*9 cells using Fugene HD reagent (Promega). 72 h post-transfection and when robust fluorescence could be observed, supernatant was collected, diluted two-fold in medium with 5%-final concentration FBS (Labtech), and sterile filtered. This virus was either used as stock directly or for passage of virus in suspension cultures. In this case only a single passage of virus was performed by infecting cells at $1x10^6$/ml with 1/100 of the virus stock. Expression cultures were inoculated with cells that had been infected with virus stock ~72 h previously. All *Sf*9 cells were grown in Insect-XPRESS (Lonza) protein-free medium with L-glutamine without additional supplements.

SEC-MALS

100 µl purified complex at 1-2 mg/ml was autoinjected onto a Superose 6 increase 10/300gl column using an Agilent 1200 series LC system. Light scattering measurements were performed using an on-line Dawn Helios ii system (Wyatt) with a QELS+ module (Wyatt) and an Optilab rEX differential refractive index detector (Wyatt). The solvent had a refractive index of 1.334 and viscosity of $9.3x10^{-3}$. The molecular mass was determined using a Zimm plot in ASTRA software (Wyatt).

Mmi1 YTH domain expression and purification

The Mmi1 ORF was gene synthesized (Thermo Fisher Scientific) to optimize codons for expression in *E. coli.* The Mmi1 sequence encoding residues 282–488 was amplified by PCR and inserted into a modified pET28a+ vector, resulting in a construct with an N-terminal hexahistidine tag with 3C protease cleavage site. Protein was expressed in *E. coli* BL21 Star (DE3) cells (Thermo Fisher Scientific) overnight at 18°C following induction with 1 mM isopropyl-β-D-thiogalactopyranoside (IPTG). Cells were lysed by sonication in 50 mM PIPES pH 7.0, 1.0 M NaCl, 5% (w/v) glycerol, 1 mM TCEP, protease inhibitor cocktail (Roche) and 0.5 mM phenylmethylsulfonyl fluoride (PMSF). Cleared lysate was batch bound to Ni-NTA agarose resin (Qiagen), washed in lysis buffer and then eluted in buffer containing 20 mM Bis-Tris pH 6.0, 300 mM NaCl, 250 mM Imidazole, 5% (w/v) glycerol and 1 mM TCEP. The His tag was cleaved overnight at 4°C with 1 mg 3C protease. Cleaved protein was diluted two-fold in 20 mM Bis-Tris pH 6.0 and 0.5 mM TCEP buffer before loading on a 5 ml HiTrap S (GE Healthcare) cation exchange column. Protein was eluted using a gradient of 0.15–1 M NaCl over 12 column volumes. Fractions containing Mmi1 were loaded onto a Superdex75 26/60pg size-exclusion column equilibrated with 20 mM PIPES pH 7.0, 150 mM NaCl and 0.5 mM TCEP.

Supplemental References

Lorenz, R., Bernhart, S.H., Höner Zu Siederdissen, C., Tafer, H., Flamm, C., Stadler, P.F., Hofacker, I.L., 2011. ViennaRNA Package 2.0. Algorithms Mol Biol 6, 26. doi:10.1186/1748-7188-6-26

Schreieck, A., Easter, A.D., Etzold, S., Wiederhold, K., Lidschreiber, M., Cramer, P., Passmore, L.A., 2014. RNA polymerase II termination involves C-terminal-domain tyrosine dephosphorylation by CPF subunit Glc7. Nat. Struct. Mol. Biol. 21, 175–179. doi:10.1038/nsmb.2753

Waterhouse, A.M., Procter, J.B., Martin, D.M.A., Clamp, M., Barton, G.J., 2009. Jalview Version 2--a multiple sequence alignment editor and analysis workbench. Bioinformatics 25, 1189–1191. doi:10.1093/bioinformatics/btp033
